# TRIM32 affects the recovery of motor function following spinal cord injury through regulating proliferation of glia

**DOI:** 10.18632/oncotarget.17492

**Published:** 2017-04-27

**Authors:** Qiang Fu, Ming-Ming Zou, Jian-Wei Zhu, Yan Zhang, Wen-Jin Chen, Mei Cheng, Chun-Feng Liu, Quan-Hong Ma, Ru-Xiang Xu

**Affiliations:** ^1^ Affiliated Bayi Brain Hospital, P.L.A. Army General Hospital, Beijing 100700, China; ^2^ Department of Neurosurgery, The 251st Hospital of P.L.A., Zhangjiakou 075000, China; ^3^ Third Military Medical University, Chongqing 400038, China; ^4^ Southern Medical University, Guangzhou 510515, China; ^5^ School of Nursing, Binzhou Medical University, Yantai 264000, China; ^6^ Institute of Neuroscience and Jiangsu Key Laboratory of Translational Research and Therapy for Neuro-Psycho-Diseases, Soochow University, Suzhou 215021, China; ^7^ Department of Neurology and Suzhou Clinical Research Center of Neurological Disease, The Second Affiliated Hospital of Soochow University, Suzhou 215004, China

**Keywords:** spinal cord injury, tripartite motif protein 32, axonal regeneration, glia, neuroinflammation

## Abstract

Both the extrinsic environmental factors and intrinsic neuronal mechanisms limit the axonal regeneration after spinal cord injury (SCI). However, the underlying molecular mechanisms remain unclear. In the present study, we identify tripartite motif protein 32 (TRIM32), an E3 ubiquitin ligase, which is barely detected in glial cells in the normal uninjured spinal cord, exhibits strong expression in both astrocytes and microglia following SCI. We further observe that deficiency of TRIM32 results in increased numbers of astrocytes and microglia, which is accompanied by enhanced proliferation of both cells and increased secretion of interleukin (IL)-1 and IL-10. The axonal regeneration is impaired in the spinal cord of TRIM32^−/−^ mice following SCI, which is indicated by increased distances of the corticospinal tracts (CST) fiber to the lesion site and less axonal sprouting. We further show that deficiency of TRIM32 results in delay motor recovery following SCI. Therefore, TRIM32 is a novel essential positive factor modulating axonal regeneration and the recovery of motor function following SCI, possibly through suppressing proliferation of glial cells.

## INTRODUCTION

Spinal cord injury (SCI), lead to death of neurons, axonal damage and demyelination, eventually resulting in permanent motor, sensory and autonomic dysfunction, which is characterized by high morbidity and disability. In general, primary spinal cord injury refers to a trauma impact on the spinal cord at the time of injury resulted in immediate irreversible tissue destruction and necrosis [1]. In addition, hours to days later, nearby surviving tissue become vulnerable immediately, triggering a cascade of pathophysiological processes including biochemical vascular response and inflammation, which further promotes tissue degeneration leading to neurological dysfunction (referred to as secondary spinal cord injury) [2]. Reduction of these secondary pathophysiological events may contribute to functional recovery following SCI, and thus as one of potential prime strategies for repairing SCI. However, the molecular mechanisms underlying the pathophysiological processes of SCI remain unclear.

Tripartite motif protein 32 (TRIM32), a member of TRIM family, is reported to possess E3 ubiquitin ligase activity [3, 4]. However, TRIM32 is also expressed in the nuclei, where it regulates transcription of genes [5–7]. TRIM32 plays essential roles in cell differentiation and proliferation, antiviral response, oncogenesis and apoptosis [8]. TRIM32 is expressed in neural progenitor cells in the developing brain and regulates neuronal differentiation [5–7, 9]. TRIM32 also has a diverse range of functions in many neurological diseases. TRIM32 expressed with high level in AD patient's occipital lobes [10]. TRIM32 regulates transcription of alpha-synuclein (snca), a risk gene in Parkinson's disease [11]. TRIM32^−/−^ mice exhibit depressive behavior when being treated by chronic stress [12]. These lines of evidence indicate that TRIM32 is important for maintenance of the physiological function of nervous system. It is worth noting that expression of TRIM32 is upregulated in the sciatic nerve upon injury [13], suggesting a potential role of TRIM32 in neural regeneration. In the present study, we observe that TRIM32, which is barely detected in glia in the normal uninjured spinal cord, is highly expressed in astrocytes and microglia of the injured spinal cord. TRIM32^−/−^ mice, following SCI, exhibit increased densities of astrocytes and microglia and elevated levels of interleukin-1 (IL-1) and IL-10, accompanied by enhanced proliferation of astrocytes and microglia at the lesion site. We further show that TRIM32^−/−^ mice exhibit slower axonal regeneration and functional recovery in locomotors. Therefore, we present that deficiency of TRIM32 results in impairment in axonal regeneration and thus functional recovery after SCI through modulating proliferation of glial cells.

## RESULTS

### Expression of TRIM32 in the spinal cord upon injury

**Figure 1:**
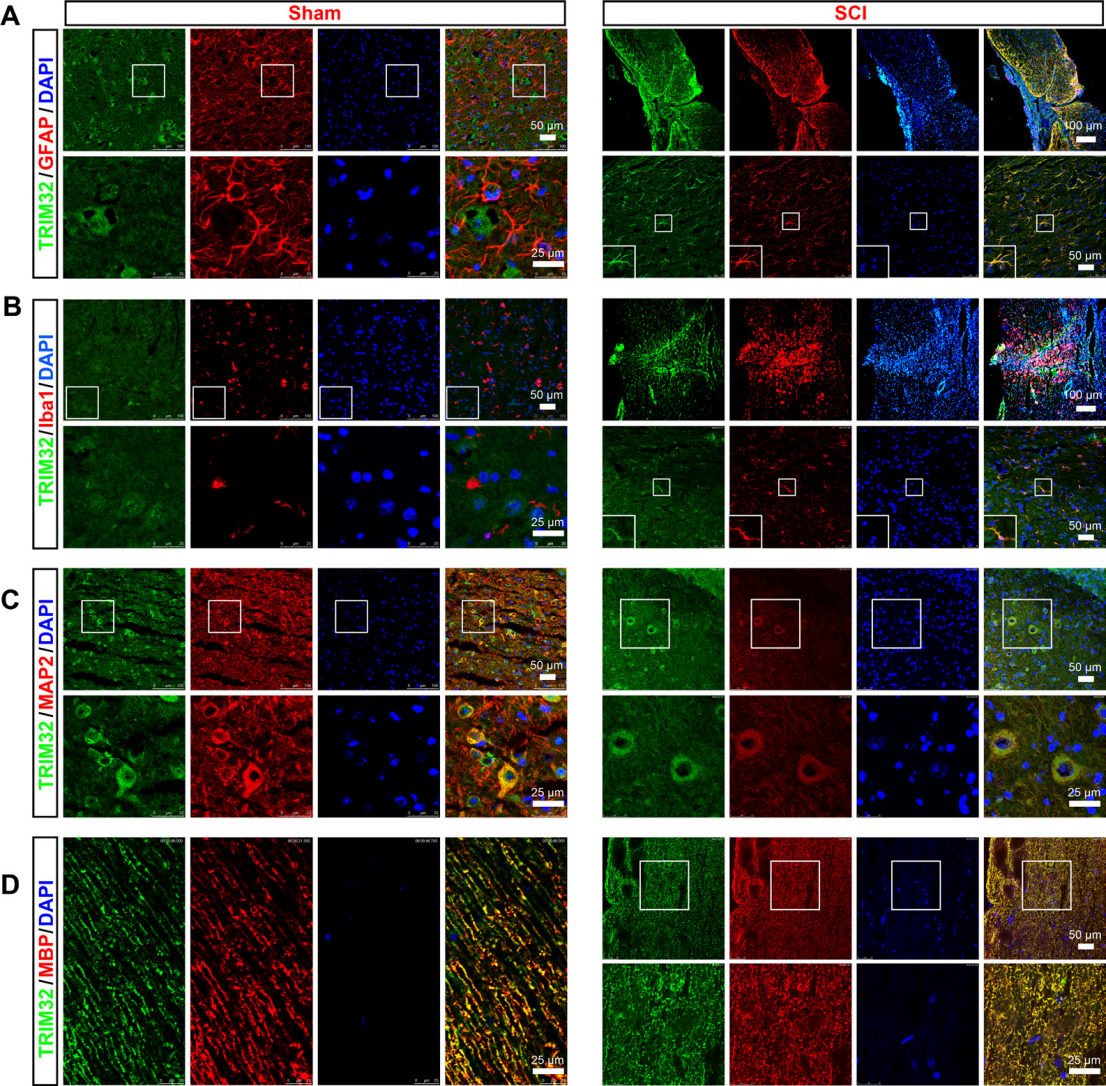
Expression of TRIM32 in the injured spinal cord The sagittal sections of spinal cord around lesion site of 4 month-old mice following SCI for 7 days or of the mice under sham surgery (Sham) were stained for TRIM32 (**A**–**D**) GFAP (A) Iba1 (B) MAP2 (C) MBP (D) and DAPI. Scale bars: 50 or 100 μm in the images with lower magnification; 25 μm in the images with higher magnification.

We first examined expression of TRIM32 in the spinal cord upon injury. The sagittal sections of the spinal cord of adult mouse following SCI or sham surgery for 14 days were coimmunostained for TRIM32 and markers for different cell types including GFAP (a marker of astrocytes; Figure 1A), Iba-1 (a marker of microglia; Figure 1B), MAP2 (a marker of neuron; Figure 1C), MBP (a marker of oligodendrocyte; Figure 1D). Expression of TRIM32 was detected in neurons (Figure 1C) and myelin sheath (Figure 1D) of both normal and injured spinal cords. It is worth noting that although TRIM32 is highly expressed in neurons (Figure 1C), few TRIM32 was observed in the myelinated axons in the spinal cord (Figure 1D). In the normal spinal cords, few TRIM32 was detected in astrocytes and microglia. In contrast, strong expression of TRIM32 was observed in astrocytes (Figure 1A) and microglia (Figure 1B), especially around the lesion site, in the injured spinal cord. These results indicate TRIM32 is upregulated by astrocytes and microglia in the spinal cord upon injury.

### Deficiency of TRIM32 results in increased numbers of neurons in the injured spinal cord

**Figure 2:**
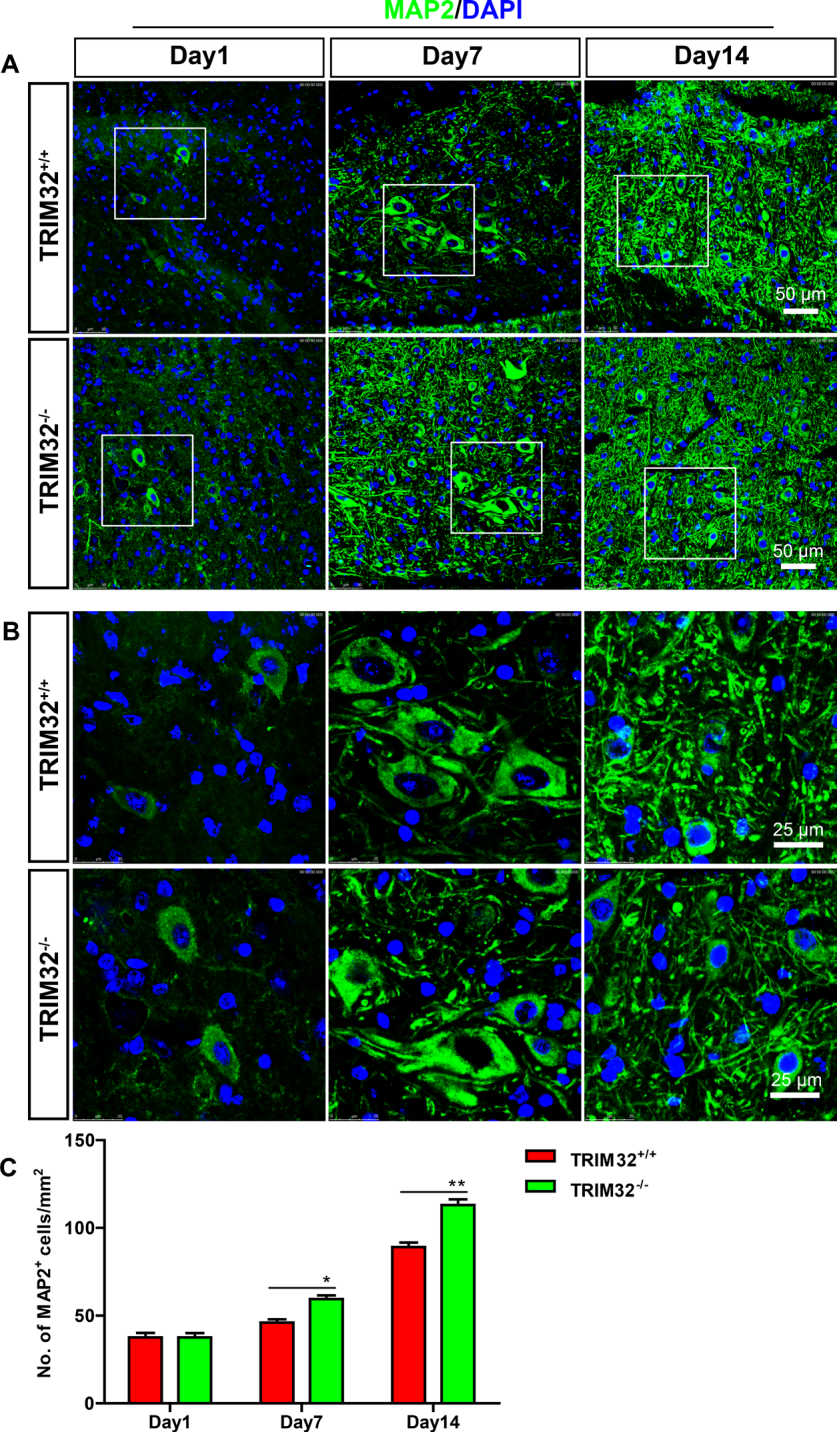
Increased number of neurons was detected in TRIM32^−/−^ mice following spinal cord injury (**A**, **B**) The sagittal sections of spinal cords of TRIM32^−/−^ and TRIM32^+/+^ mice around lesion sites following SCI for 1, 7 and 14 days were stained for MAP2 and DAPI. Images with higher magnification were shown in B. Scale bars: 50 μm (A) 25 μm (B) (**C**) MAP2^+^ density was quantified and expressed as the numbers of MAP2^+^ cells per area. Data were presented as mean + SEM. *n* = 40 sections in 8 mice/genotype. *P < 0.05; **P < 0.01.

TRIM32 regulates proliferation, neuronal differentiation of neural stem cells [5–7, 9]. We thus examined whether deficiency of TRIM32 results in abnormal numbers of neurons in the injured spinal cord. The sagittal sections of the spinal cords of TRIM32^+/+^ and TRIM32^−/−^ littermates, which were undergone SCI for 1, 7 and 14 days, were immunostained for MAP2 (Figure 2A, 2B). The numbers of MAP2^+^ cells in the same distance from the lesion site were quantified. Increased numbers of MAP2^+^ cells were observed in the spinal cords of TRIM32^−/−^ mice following SCI for 7 and 14 days, compared to those in TRIM32^+/+^ littermates (Figure 2C). These results indicate that deficiency of TRIM32 leads to increased numbers of neurons in the injured spinal cord.

### Deficiency of TRIM32 results in increased numbers of astrocytes and microglia in the injured spinal cord

**Figure 3:**
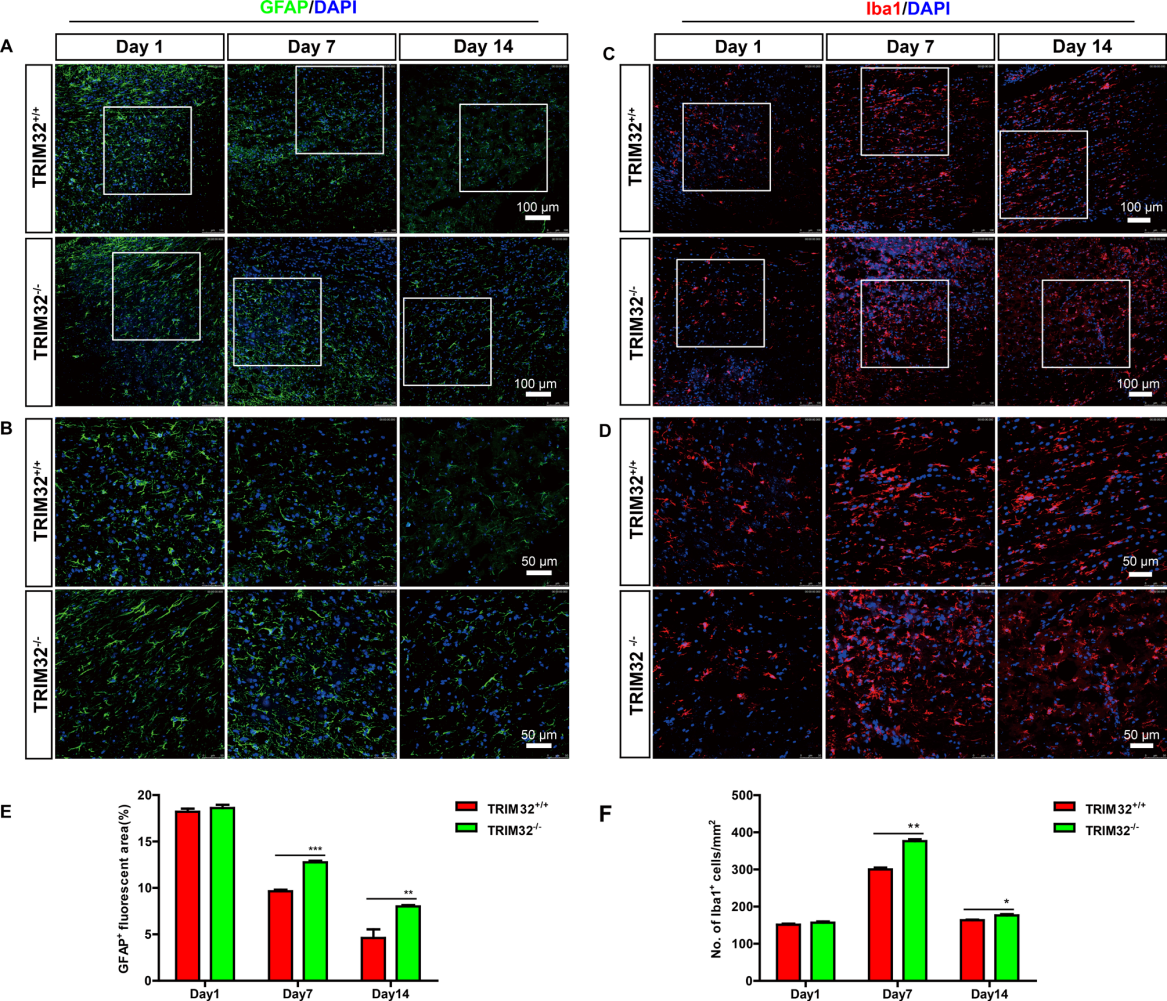
TRIM32^−/−^ mice exhibited increased numbers of astrocytes and microglia following spinal cord injury (**A**–**D**): The sagittal sections of spinal cords of TRIM32^−/−^ and TRIM32^+/+^ mice around lesion sites following SCI for 1, 7 and 14 days were stained for GFAP (A) or Iba-1 (C) and DAPI. Images with higher magnification were shown in B, D. Scale bars: 100 μm (A, C) 50 μm (B, D). (**E**, **F**) GFAP^+^ (E) or Iba-1^+^ (F) density were quantified and expressed as the percentage of marker^+^ area per total area. Data were presented as mean + SEM. *n* = 4 0 sections in 8 mice/genotype. **P < 0.01; ***P < 0.001.

We further examined whether deficiency of TRIM32 results in abnormal numbers of glia in the injured spinal cords. The sagittal sections of the spinal cords of TRIM32^+/+^ and TRIM32^−/−^ littermates following SCI for 1, 7 and 14 days, were immunostained for GFAP (Figure 3A, 3B) and Iba1 (Figure 3C, 3D). Similar to neurons, increased numbers of astrocytes (Figure 3E) and microglia (Figure 3F) have been observed in the spinal cords undergone SCI for 7 and 14 days, but not for 1 day. These results indicate that deficiency of TRIM32 results in increased numbers of astrocytes and microglia in the injured spinal cords.

### Deficiency of TRIM32 results in enhanced proliferation of glia in the injured spinal cords

**Figure 4:**
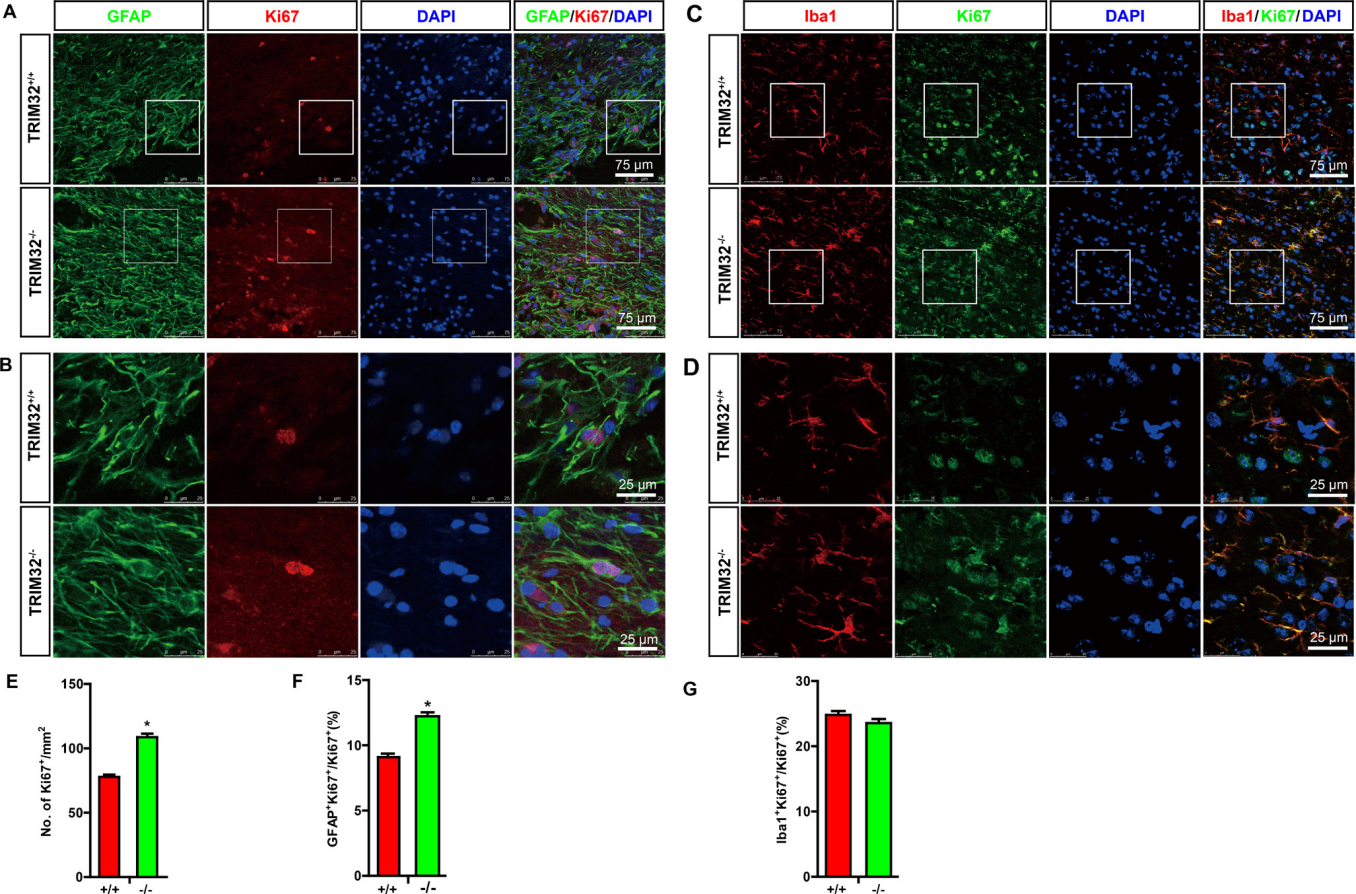
TRIM32^−/−^ mice exhibited increased proliferation of astrocytes and microglia following spinal cord injury (**A**–**D**) The sagittal sections of spinal cords of TRIM32^−/−^ and TRIM32^+/+^ mice around lesion sites following SCI for 7 days were stained for GFAP (A) or Iba-1 (C) Ki67 and DAPI. Images with higher magnification were shown in B, D. Scale bars: 75 μm (A, C) 25 μm (B, D). (**E**–**G**) The density of Ki67^+^ cells were quantified and expressed as the numbers of Ki67^+^ cells per mm^2^ (E). The density of GFAP^+^Ki67^+^ (F) or Iba-1^+^Ki67^+^ (G) cells were quantified and expressed as the percentage of total number of Ki67^+^ cells. Data were presented as mean + SEM. *n* = 30 sections in 6 mice/genotype. *P < 0.05.

Considering the fact that TRIM32 regulates proliferation of stem cells [5, 6, 9], we further asked whether the increased numbers of glial cells due to an enhanced proliferation. The sagittal sections of the spinal cords following SCI for 7 days were coimmunostained for either GFAP (Figure 4A, 4B) or Iba-1 (Figure 4C, 4D) and Ki67, a marker for active cell cycle. The numbers of Ki67^+^ cells, which represent the cells in the cell cycle, were quantified. TRIM32^−/−^ mice exhibited increased numbers of Ki67^+^ cells in the injured spinal cords, compare to TRIM32^+/+^ littermates (Figure 4E), indicating that deficiency of TRIM32 results in enhanced proliferation in the injured spinal cords. Moreover, the numbers of GFAP^+^Ki67^+^ cells increased in the injured spinal cords of TRIM32^−/−^ mice, compared to those in TRIM32^+/+^ littermates (Figure 4F), indicating that deficiency of TRIM32 results in enhanced proliferation of astrocytes in the injured spinal cords. Similarly, increased numbers of Iba-1^+^Ki67^+^ have been observed in the spinal cords of TRIM32^−/−^ mice following SCI, compared to those in TRIM32^+/+^ littermates (Figure 4G), indicating deficiency of TRIM32 results in enhanced proliferation of microglia in the injured spinal cord. Therefore, there results indicate that deficiency of TRIM32 promotes proliferation of astrocytes and microglia in the spinal cord upon injury.

### Deficiency of TRIM32 results in increased secretion of cytokines

**Figure 5:**
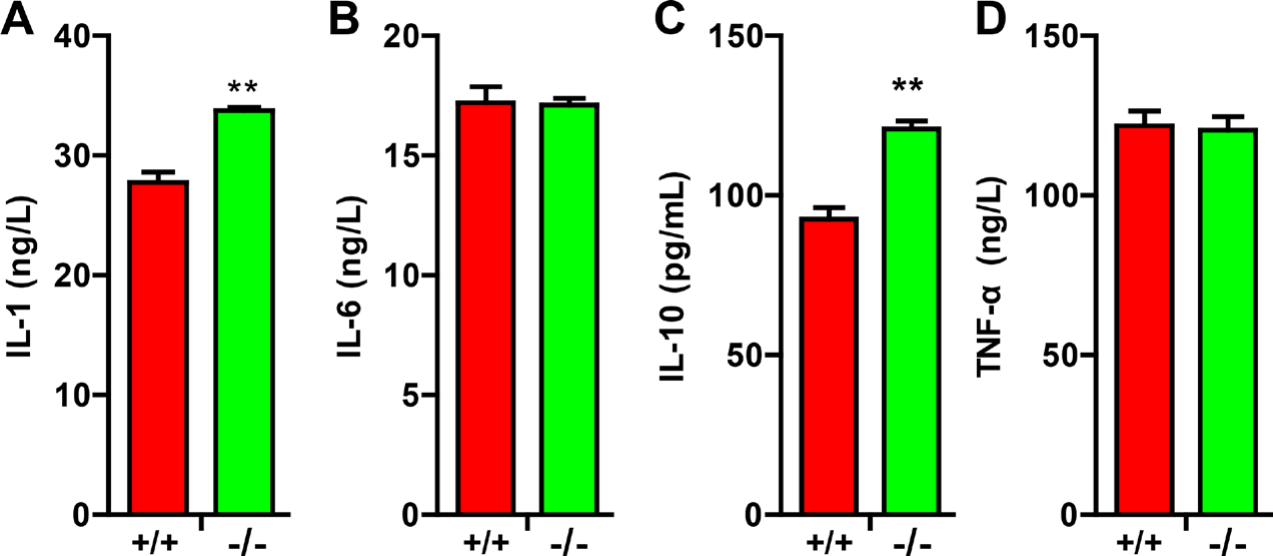
TRIM32^−/−^ mice exhibited elevated cytokines following spinal cord injury (**A**–**D**) ELISA analysis of the levels of IL-1 (A) IL-6 (B) IL-10 (C) and TNF-α (D) in the spinal cords of TRIM32^−/−^ and TRIM32^+/+^ mice following SCI for 14 days. Data were presented as mean+SEM. *n* = 6 mice/genotype. **P < 0.01.

Activated astrocytes and microglia through secreting cytokines modulates the pathophysiology of various disorders such as neurodegenerative disease, traumatic brain injury and SCI [14]. We thus examined the levels of cytokines in TRIM32^−/−^ mice following SCI. ELISA analysis showed that the levels of IL-1 (Figure 5A) and IL-10 (Figure 5C) increased in the spinal cords of TRIM32^−/−^ mice, which were following SCI for 14 days, compared to those in TRIM32^+/+^ mice. In contrast, IL-6 (Figure 5B) and tumor necrosis factor-alpha (TNF-α) (Figure 5D) exhibited similar levels in the spinal cords of TRIM32^−/−^ and TRIM32^+/+^ mice following SCI. Thus, these results indicate that deficiency of TRIM32 results in increased levels of IL-1 and IL-10.

### Deficiency of TRIM32 results in impaired neural regeneration and recovery of motor functions following SCI

**Figure 6:**
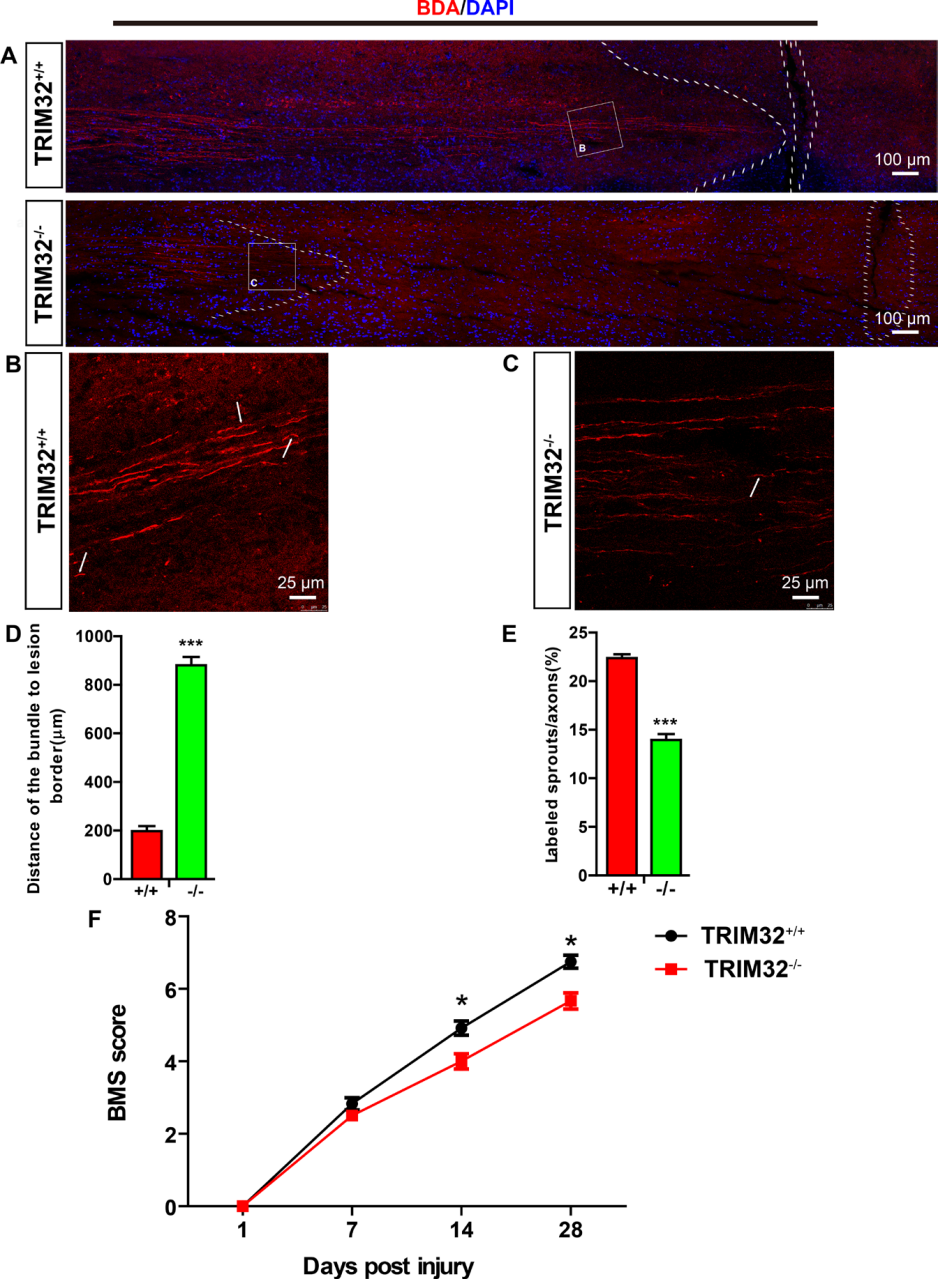
TRIM32^−/−^ mice exhibited impaired axonal regeneration and behavioral recovery following spinal cord injury (**A**–**C**) BDA-labeled CST in proximity of the lesion sites of TRIM32^−/−^ and TRIM32^+/+^ mice following SCI for 28 days. Images with higher magnification were shown in B and C. Scale bars: 100 μm (A) 25 μm (B, C). (**D**) The distance of the sprouting axons to the lesion sites was quantified. (**E**) The numbers of sprouting axons were quantified and expressed as the percentage of total labeled CST. (**F**) BMS scores of TRIM32^−/−^ and TRIM32^+/+^ mice following SCI at day 1, day 7, day 14 and day 28.

The reorganization and the formation of new circuits which were due to synaptic plasticity and collateral sprouting of lesioned and unlesioned descending tracts, determine the functional recovery from SCI. We thus examined the role of TRIM32 on axonal sprouting of the corticospinal tracts (CST). The cortical neurons in layer V were labeled by BDA tracer injection in the sensorimotor cortex. The CST sprouting and retraction bulb indexes at the spinal cord level were calculated. The distances of CST to the lesion site increased in TRIM32^−/−^ mice following SCI for 14 days, compared to those in TRIM32^+/+^ mice (Figure 6A, 6D), indicating an enhanced dieback of the CST. Moreover, TRIM32^−/−^ mice exhibited decreased percentages of sprouting axons in the CST (Figure 6B, 6C, 6E), indicating a retarded axonal regeneration. Thus, deficiency of TRIM32 results in impaired axonal regeneration upon injury in the spinal cord.

Next, we examined whether slow regeneration in TRIM32^−/−^ spinal cord following injury led to poor functional recovery. The locomotor recovery was evaluated using the Basso Mouse Scale for Locomotion (BMS) for 28 days after SCI [15]. Both TRIM32^−/−^ and TRIM32+/+ littermates became almost complete paraplegia, with extremely poor ankle movement, right after the bilateral dorsal cut hemisection injury. Both mice exhibited locomotor recovery gradually with time after SCI. However, TRIM32^−/−^ mice exhibited an overall impaired locomotor performance, with a significant decline 14 and 28 days following SCI, compared to TRIM32^+/+^ mice (Figure 6F). These results suggest that TRIM32 deficiency impairs the recovery of hind limb motor function.

## DISCUSSION

Both the extrinsic environmental factors and intrinsic neuronal mechanisms limit the axonal regeneration after SCI. In the present study, we show that TRIM32, an E3 ubiquitin ligase, is an essential regulator in axonal regeneration after SCI. Deficiency of TRIM32 results in impaired axonal regeneration and thus functional recovery after SCI, which is accompanied by increased numbers of astrocytes and microglia. In the normal spinal cord, TRIM32 is not expressed in glial cells. However, upon injury, both astrocytes and microglia, which are activated around the lesion site, express high levels of TRIM32. The re-expression of TRIM32 in glia upon injury suggests that TRIM32 play an important role in regulating glia upon injury. Consistent with this idea, we observe that deficiency of TRIM32 results in increased numbers of astrocytes and microglia through enhanced proliferation of both cells. We also observe increased numbers of neurons in the injured spinal cords of TRIM32^−/−^ mice. However, such increased numbers of neurons fail to enhance axonal regeneration in the injured spinal cord. Moreover, it should note that although TRIM32 is expressed by neurons, few TRIM32 is detected in myelinated axons. Therefore, the impaired axonal regeneration in the injured spinal cord of TRIM32^−/−^ mice may due to the consequence of increased numbers of glial cells.

Cell cycle activation in glial cells is a crucial event that contributes to secondary damage after SCI such as activation of astrocytes and microglia [16]. TRIM32 inhibits proliferation of embryonic and adult neural stem cells [6, 9, 17]. In contrast, several studies indicate that TRIM32 is an oncogene, which promotes proliferation of cancer cells [18, 19]. We here show a novel role of TRIM32 in regulating proliferation of glial cells in the spinal cord upon injury. These studies indicate that the functions of TRIM32 in regulation of cell proliferation are context-dependent. Considering the fact that TRIM32 is not expressed by glial cells in the normal spinal cord, TRIM32-regulating proliferation of glial cells is a process occurred upon injury.

Astrocytes, the most abundant glial cells in the central nervous system, plays a variety of roles in the homeostasis of the CNS [14]. However, upon injury, astrocytes upregulate expression of astroglial genes such as GFAP, vimentin, and nestin [20], and exhibit a proliferative phenotype to form a dense and impermeable glial scar around the lesion [21]. At early stages of SCI, the formation of glial scar serves as a defense mechanism through isolating from the neurotoxicity, limiting the immune cells into the injured spinal cord and preventing the spread of secondary degeneration [22], thus controlling and repairing the initial damage. However, as time elapses, it ultimately results in some harmful effects, biochemically and physically inhibiting axonal outgrowth. For example, chondroitin sulphate proteoglycans (CSPGs), expressed in the extracellular matrix of astrocytes, upregulates its levels after injury [23]. CSPGs within the glial scar and perineuronal net potently create a barrier to impede axonal regrowth and sprouting [24]. However, some CSPGs such as CSPG4 and CSPG5 support axon growth, which are upregulated in the astrocytes in the injured spinal cord. This is also consistent with the recent finding that axon fails regenerate when complete deletion of astrocytic scars in the injured spinal cord [25]. In addition, activated astrocytes release small molecules to maintain inflammatory reaction and to regulate the activity of other cells such as microglia. Consistently, competing studies have shown that decreased gliosis will improve axonal regeneration and functional recovery [26–29]. Thus, deficiency of TRIM32 results in impaired axonal regeneration may due to glial scars formed by increased astrocytes.

We also observe upregulation of IL-1 and IL-10 in the spinal cord of TRIM32^−/−^ mice. IL-1 plays an important role in secondary injury after SCI through enlarging the lesion sizes and inducing apoptosis of neuronal cells [30, 31]. Consistent with these observations, IL-1 receptor antagonist treatment reduced the contusion-induced apoptosis and the lesion size [32]. IL-10, a key indicator of M2 macrophage activation, mediates anti-inflammatory response and enhances neuronal survival following spinal cord injury [33]. Some studies indicate that expression of IL-10 upon injury is induced by astrocytes and microglia [34]. Therefore, it seems that increased numbers of astrocytes and microglia in the injured spinal cord of TRIM32^−/−^ mice upregulates IL-10, which enhances the numbers of neurons through promoting neuronal survival. However, such “neuronal protection” function fails to compensate the inhibitory function of neuroinflammtion mediated by increased proliferation of astrocytes and microglia.

TRIM32 is an E3 ubiquitin ligase, involved in degradation of protein through the proteasome. However, TRIM32 is also detected in the nuclei, where it mediates transcription of genes. We here observe a strong function of TRIM32 in regulation of proliferation of glial cells. Since most expression of TRIM32 is detected in the cytoplasm of glial cells (Figure 1A, 1B), we suppose that TRIM32 may regulates proliferation of glial cells through a mechanism involved in protein degradation. However, it remains to be further investigated the molecular mechanisms underlying that TRIM32 regulates proliferation of glial cells.

In conclusion, the present study identifies that TRIM32 is an essential modulator in mediating axonal regeneration and thus recovery of motor functions following SCI, which is linked with regulation of proliferation of glial cells.

## MATERIALS AND METHODS

### Mice

Mice deficient in TRIM32 (TRIM32^−/−^ mice) have been described [35]. They were derived from heterozygous breeding pairs, and wild-type (TRIM32^+/+^) littermate mice were used as controls. Female mice at 8 weeks old of age were used in this study. All mice were handled according to the protocols approved by the Institutional Animal Care and Use Committee of Soochow University and Beijing Military Hospital.

### Antibodies

Mouse ionized calcium binding adaptormolecule-1 (Iba-1) antibody (Wako, 016-26461), Mouse Glial Fibrillary Acidic Protein antibody (GFAP) (Millipore, Mab360), Mouse Microtubule Associated Protein 2 (MAP2) antibody (Abcam, ab11267), Mouse myelin basic protein antibody (MBP) (Santa Cruz, sc-66064), Rabbit TRIM32 antibody (Santa Cruz, sc-99011), Mouse TRIM32 antibody (Sigma, SAB1407164), Rabbit Ki67 antibody (Abcam, ab16667), Mouse Ki67 antibody (Abcam, ab8191). The corresponding fluorescent secondary antibodies were form Invitrogen.

### Surgical procedure

Animals were anesthetized by intraperitoneally injection of with 3.6% chloral hydrate (0.01 L/kg). Approximately 10 mm of spine was exposed at T8, and the mice were subjected to a bilateral dorsal cut hemisection using iridectomy scissors to transect the left and right dorsal funiculus, the dorsal horns, and the ventral funiculus. Body temperature was maintained by keeping the mice on a heating pad (37°C) during the whole surgical procedure. After surgery, mice were placed back in their cages warmed up with an infrared light to prevent hypothermia.

### Behavioral analysis

All animals received training before surgery. During training, the mice were exposed to the open field for the locomotor test twice a day, each 3 min, for at least 5 days. Mice were tested 1, 7, 14, 21 and 28 d following injury. Each mouse was allowed to freely move in the open field for 3 min. The open-field locomotion was assessed using Basso Mouse Scale (BMS) as described [15].

### BDA tracing

Biotinylated dextran amine (BDA) (Invitrogen) was injected to the right sensorimotor cortex at two weeks following the injury. The tracer was injected at four sites (0.4 μl per site over a period of 5 min, plus 3 min of the glass capillary in place to avoid spillover), coordinates (from bregma) were 1.0 mm lateral, 0.5 mm deep, and +0.5, **−**0.2, **−**0.7, and −1 mm. The mice were sacrificed two weeks after injection.

### Immunofluorescent staining

The mice were perfused with 4% paraformaldehyde cardically and were postfixed in 4% paraformaldehyde for overnight followed by being cryoprotected in 30% sucrose for at least 48 h at 4°C. Spinal cords were sectioned sagittally on a cryostat at 10 μm in thickness. Sections were washed 3 times with PBS containing 0.3% Triton X-100 (PBST), 10 min each, followed by blocking with 10% BSA in PBS for 1 h, and incubated with primary antibody overnight at 4°C. Sections were washed 3 times in PBST and incubated with appropriate secondary Alexafluor-conjugated antibodies for 2 h at room temperature. The sections were then washed 3 times with PBST and mounted in mounting medium containing DAPI (Vector Laboratories, Burlingame). The stained sections were examined by a confocal laser-scanning microscope (LeicaTCSSP5II) with 10× lens (506511), 20× lens (506513) and 40× lens (506295) respectively.

### Enzyme-linked immunosorbent assay

The T7–T9 spinal cord was rapidly removed and snapped frozen in liquid nitrogen. The levels of IL-1, IL-6, IL-10 and TNF-α were quantified by enzyme-linked immunosorbent assay kit (Beijing biolab Technology Co., Ltd) according to the manufacturer's instructions.

### Image analysis

Ten to eleven sagittal sections each spinal cord and four mice per group were analyzed. Sections matched in anatomy were serially chosen. The density of glia cells and neurons in the rostral area 400 μm away from the lesion border was analyzed. The area of GFAP^+^ cells in each image were quantified by Image J (National Institutes of Health, Bethesda, MD, USA) as described [36]. The densities of GFAP^+^ cells were quantified as the area that was occupied by GFAP^+^ signals divided by total area of the spinal cord. The densities of MAP2^+^ cells and IBA-1^+^ cells were quantified and expressed as the numbers of marker^+^ cells divided by the area of outlined regions. 100 μm^2^ area per image was selected randomly for quantification. Five slices per mouse and 8 mice per genotype were quantified.

### Statistical analysis

Data were presented as means ± SEM. Statistical analysis was performed using SPSS18.0 software. Two-tailed Student's *t-test* and repeated-measurement ANOVA was used. Significance was accepted at *P* < 0.05. *P < 0.05, **P < 0.01, ***P < 0.001.

## SUPPLEMENTARY FIGURES



## Abbreviations

ASD: Autism spectrum disorder
BMS: Basso Mouse Scale
BDA: Biotinylated dextran amine
CSPGs: chondroitin sulphate proteoglycans
CST: corticospinal tracts
GFAP: Glial Fibrillary Acidic Protein
IL: interleukin
MAP2: Microtubule Associated Protein 2
MBP: myelin basic protein
SCI: spinal cord injury
TRIM32: tripartite motif protein 32
TNF-α: tumor necrosis factor-alpha
