# Supplementary material for: TRIM32 affects the recovery of motor function following spinal cord injury through regulating proliferation of glia
Source: Oncotarget. 2017 Apr 27;8(28):45380–90. doi: 10.18632/oncotarget.17492 (PMC5542194; doi:10.18632/oncotarget.17492)
Supplement: Supplementary file 1 [file oncotarget-08-45380-s001.pdf]

TRIM32 affects the recovery of motor function following spinal cord injury through regulating proliferation of glia

Supplementary Materials

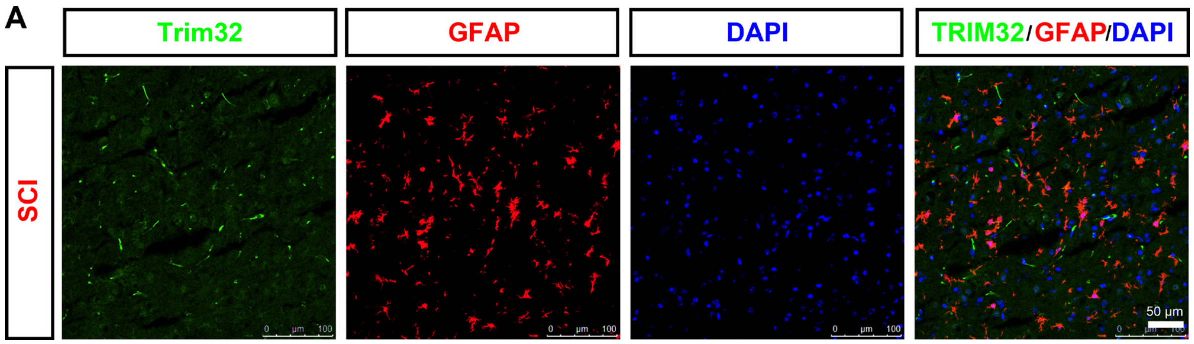

Supplementary Figure 1: Expression of TRIM32 in the injured spinal cord of TRIM32$^{-/-}$ mice. The sagittal sections of spinal cord around lesion site of 4 month-old mice following SCI for 7 days or of TRIM32$^{-/-}$ mice under sham surgery (Sham) were stained for TRIM32 and GFAP. Scale bars: 50 µm.

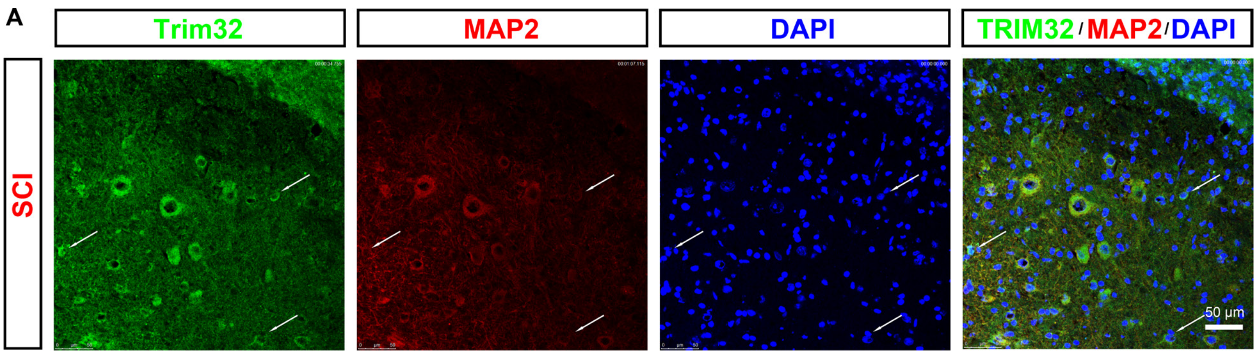

Supplementary Figure 2: Expression of TRIM32 in the injured spinal cord. The sagittal sections of spinal cord around lesion site of 4 month-old mice following SCI were stained for TRIM32 and MAP2. The arrows indicate MAP2^{-}TRIM32^{+} cells. Scale bars: 50 µm.
